# Inhibition of histone H3K27 demethylases selectively modulates inflammatory phenotypes of natural killer cells

**DOI:** 10.1074/jbc.RA117.000698

**Published:** 2018-01-04

**Authors:** Adam Cribbs, Edward S. Hookway, Graham Wells, Morten Lindow, Susanna Obad, Henrik Oerum, Rab K. Prinjha, Nick Athanasou, Aneka Sowman, Martin Philpott, Henry Penn, Kalle Soderstrom, Marc Feldmann, Udo Oppermann

**Affiliations:** From the ‡ Botnar Research Center, Nuffield Department of Orthopedics, Rheumatology and Musculoskeletal Sciences, National Institute of Health Research Oxford Biomedical Research Unit (BRU), University of Oxford, Oxford OX3 7DQ, United Kingdom,; the § Kennedy Institute of Rheumatology Nuffield Department of Orthopedics, Rheumatology and Musculoskeletal Sciences, National Institute of Health Research Oxford BRU and; the ‡‡ Structural Genomics Consortium, University of Oxford, Oxford OX3 7LD, United Kingdom,; the ¶ Roche Innovation Center Copenhagen A/S, DK 2970 Hørsholm, Denmark,; the ‖ Epinova Discovery Performance Unit, Medicines Research Centre, GlaxoSmithKline R&D, Stevenage SG1 2NY, United Kingdom,; the ** Arthritis Centre, Northwick Park Hospital, Harrow, HA13UJ, United Kingdom,; the §§ Freiburg Institute of Advanced Studies, 79104 Freiburg, Germany, and; the ¶¶ Oxford Centre for Translational Myeloma Research Oxford, Oxford OX3 7DQ, United Kingdom

**Keywords:** histone demethylase, histone modification, enzyme inhibitor, epigenetics, natural killer cells (NK cells), inhibitor

## Abstract

Natural killer (NK) cells are innate lymphocytes, important in immune surveillance and elimination of stressed, transformed, or virus-infected cells. They critically shape the inflammatory cytokine environment to orchestrate interactions of cells of the innate and adaptive immune systems. Some studies have reported that NK cell activation and cytokine secretion are controlled epigenetically but have yielded only limited insight into the mechanisms. Using chemical screening with small-molecule inhibitors of chromatin methylation and acetylation, further validated by knockdown approaches, we here identified Jumonji-type histone H3K27 demethylases as key regulators of cytokine production in human NK cell subsets. The prototypic JMJD3/UTX (Jumonji domain–containing protein 3) H3K27 demethylase inhibitor GSK-J4 increased global levels of the repressive H3K27me3 mark around transcription start sites of effector cytokine genes. Moreover, GSK-J4 reduced IFN-γ, TNFα, granulocyte–macrophage colony-stimulating factor (GM-CSF), and interleukin-10 levels in cytokine-stimulated NK cells while sparing their cytotoxic killing activity against cancer cells. The anti-inflammatory effect of GSK-J4 in NK cell subsets, isolated from peripheral blood or tissue from individuals with rheumatoid arthritis (RA), coupled with an inhibitory effect on formation of bone-resorbing osteoclasts, suggested that histone demethylase inhibition has broad utility for modulating immune and inflammatory responses. Overall, our results indicate that H3K27me3 is a dynamic and important epigenetic modification during NK cell activation and that JMJD3/UTX-driven H3K27 demethylation is critical for NK cell function.

## Introduction

Natural killer (NK)^3^ cells are group 1 innate lymphocytes and facilitate maintenance of tissue homeostasis following sensing of target cells through coordination of activating receptors, such as NKG2D, DNAM-1, NKp30, NKp46, NKp44, and inhibitory receptors such as killer immunoglobulin-like receptors and CD94-NKG2A (1–3). It is now recognized that in addition to their critical sentinel functions in providing lytic effector roles, *e.g.* through clearance of virally infected or malignant, transformed cells, NK cells contribute to inflammatory processes, as observed in autoimmune diseases such as rheumatoid arthritis (RA) (4), by shaping the inflammatory cytokine microenvironment.

NK cells are characterized by the expression of CD56, the 140-kDa isoform of neural cell adhesion molecule, and a lack of expression of cell-surface CD3 (5). The plasticity and functional heterogeneity of NK cells is not fully understood, but NK cells can be grouped into at least three subsets based upon the expression of CD56 and CD57, a known marker of replicative senescence and terminal differentiation in CD8+ T cells (6, 7). CD56^Bright^CD57^−^ NK cells express high IFN-γ levels and exert a low cytotoxic effector function when compared with CD56^dim^CD57^+^ NK cells, which express low IFN-γ and provide a high degree of cytotoxicity. A third population of NK cells, CD56^dim^CD57^−^ cells, is an intermediate population that expresses moderate levels of IFN-γ and cytotoxic effector function. Following activation, NK cells mediate cytotoxic killing of target cells through two major mechanisms that require direct contact between NK cells and their target cells (8). The first pathway involves target cell lysis mediated by cytotoxic molecules (*e.g.* perforin and granzymes) that are stored in secretory lysosomes (9). The second pathway involves the engagement of death receptors with their ligands (*e.g.* FasL or TRAIL) that results in caspase-dependent apoptosis (10). Moreover, NK cells are poised to release cytokines and growth factors that can initiate inflammatory responses mediated by both the innate and the adaptive arms of the immune system (11).

The term “epigenetics” defines potentially heritable, chromatin-templated cellular phenotypes that are independent of the underlying DNA sequence (12). More loosely, epigenetics is frequently used to describe various chromatin modification processes. Chromatin remodeling and post-translational modifications of N-terminal, unstructured tails of histone proteins are mechanisms of importance in *e.g.* embryonic development, cancer, or the immune response (13–16). Chromatin processes have now been recognized as key components in the regulation and signaling of functional states of the epigenomic landscape thereby controlling gene transcription, DNA replication, and repair (17). The dynamic nature of chromatin modification has now been realized, and recognition of specific modifications by several proteins has enabled the concept of a chromatin or “histone code” (18). Presently, several classes of histone modifications have been identified (19), and of particular importance is histone methylation, which plays a pivotal role in the maintenance of both active and suppressed states of gene expression, depending on the sites and degree of methylation (19–21). In particular, the methylation of histone H3 at lysine residues −4, −36, and −79 (H3K4, H3K36, and H3K79) is implicated in the activation of transcription, whereas methylation of histone H3 at lysine −9 and −27 (H3K9 and H3K27) is correlated with repression of transcription. Although lysine methylation was initially considered a stable modification, it is now recognized that the interplay between histone methylation and demethylation provides an important layer in tuning transcriptional responses and programs. For example, the methyltransferase EZH2 (*enhancer of zeste homolog 2*) catalyzes the *S*-adenosylmethionine–dependent trimethylation of H3K27, whereas several members of the Jumonji domain–containing (Jmj) Fe^2+^ and 2-oxoglutarate–dependent oxygenases catalyze demethylation of methylated histone lysine residues *in vitro* and *in vivo* (22–26). In particular, Jmj family members 3 (JMJD3 and KDM6B) and ubiquitously transcribed tetratricopeptide repeat gene, X chromosome (UTX and KDM6A) were shown to be specific demethylases of H3K27me2/3 (22, 23, 25). Whereas UTX appears to be constitutively expressed in many tissues, JMJD3 is inducible by lipopolysaccharide, several cytokines, and growth factors (27, 28). Both enzymes play important roles in macrophage responses (27, 29), development, and stem cell function (30). However, the enzymes appear to have dual roles, by exerting their functions as scaffolding and being catalytically active. Importantly, UTX is a component of transcriptional complexes, such as *Trithorax* and MLL (*mixed lineage leukemia*), which regulate gene activation through H3K4 methyltransferase and H3K27 demethylase activities (31).

Thus far, few studies have demonstrated a role for DNA methylation in regulation of NK cell IFN-γ secretion (32–34) and for histone H3K27 methylation by EZH2 in NK cell development (35). Considering the importance of epigenomic regulation in immune cell function (36) and the apparent lack of chromatin regulation data on NK cell function, we set out to identify epigenetic factors involved in NK activation, leading us to investigate the role of histone H3K27 demethylation in NK cell function in further detail.

## Results

### NK cell cytokine production is regulated by inhibitors of chromatin methylation and acetylation

**Figure 1.**
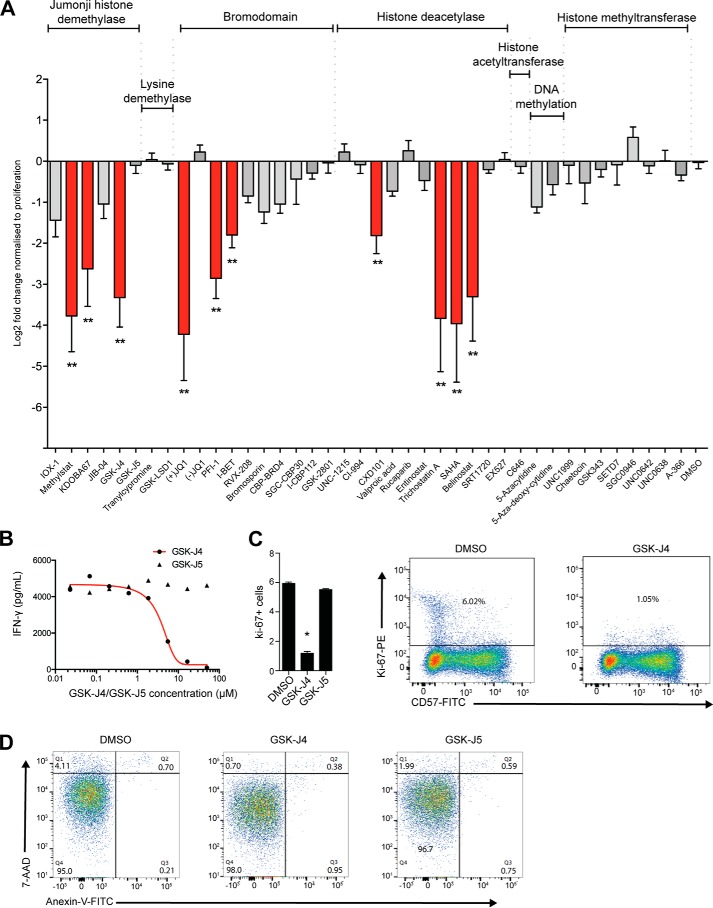
**Jumonji histone demethylase, BET bromodomain, and histone deacetylase inhibition prevent IFN-γ release in human NK cells.** *A*, NK cells were isolated from peripheral blood, stimulated with IL-15, and cultured for 48 h in the presence of DMSO or small molecule epigenetic inhibitor, and supernatant was then harvested. Levels of IFN-**γ** were measured by ELISA, and log2-fold changes were determined against DMSO following normalization against cellular proliferation as measured by an MTS assay (*n* = 9). Statistical significance was determined by ANOVA with Dunnett's post hoc test. *B*, the IC_50_ values for IFN-**γ** release for inhibitors GSK-J4 and its inactive control molecule GSK-J5 were determined by culturing different concentrations of each inhibitor with IL-15–stimulated NK cells following 48 h of culture. *C*, NK cells were isolated; stimulated with IL-15; and cultured with DMSO, GSK-J4, or GSK-J5 for 48 h; stained with CD57 and Ki-67; and measured using flow cytometry. The data are from four independent experiments. *Error bars* represent standard deviation, and statistical significance was determined by Kruskal–Wallis with Dunn's multiple comparison test. *, *p* < 0.05. *D*, NK cells were isolated from peripheral blood, stimulated with IL-15, and cultured in the presence of DMSO, GSK-J4, or GSK-J5 for 48 h. The cells were stained with annexin V–FITC and 7-AAD and then analyzed using flow cytometry. In each panel, the *lower left quadrant* shows cells that are negative for both 7-AAD and annexin V, whereas the *upper left quadrant* shows only 7-AAD–positive cells (necrotic). The *lower right quadrant* shows cells that are positive for annexin V (early apoptotic), and the *upper right quadrant* shows annexin V- and 7-AAD–positive cells (late apoptotic).

Our initial aim was to identify epigenetic pathways that are important for mediating inflammatory NK cell responses. Although NK cells secrete a number of pro-inflammatory cytokines in response to activation, the hallmark of an NK cell is its ability to secrete pro-inflammatory IFN-γ when stimulated using cytokines such as IL-2, IL-12, or IL-15 (37, 38). We used the production of IFN-γ as hallmark of NK cell function in assessing the effectiveness of screening with a focused library of small molecule inhibitors, which consisted of targeting eight major distinct mechanistic classes of chromatin and epigenetic proteins (Table S1). We first prescreened the library in the established IFN-γ producing Nishi NK-like cell line (39) (Fig. S1). These data reveal a significant reduction of secreted IFN-γ with three different classes of inhibitors targeting the JmjC domain–containing histone demethylases, histone deacetylases (HDACs), and bromodomains. We then tested a subset of the library in IL-15–stimulated primary human NK cells derived from the peripheral blood of healthy donors, again confirming strong inhibition of IFN-γ production in these three pathways (Fig. 1*A*). These results were confirmed by IC_50_ measurements for the compounds that potently inhibited IFN-γ, comprising the H3K27 demethylase inhibitor GSK-J4 (Fig. 1*B*) (16), the HDAC inhibitor suberoylanilide hydroxamic acid (vorinostat) (40) and the bromodomain and extra-terminal (BET) family proteins inhibitor +JQ1 (41) (Fig. S2). These experiments also included the use of negative control compounds, *i.e.* inactive regional isomers like GSK-J5 (16, 42), which provide additional support for a specific on-target activity of GSK-J4. We observed that culturing NK cells with GSK-J4 led to a reduction in Ki67^+^ NK cells, indicating that GSK-J4 may have a role in regulating proliferation (Fig. 1*C*). Because reduced NK cell inflammatory function may also be indicative of increased malfunction or even cell death, we next investigated the cytotoxic effect of GSK-J4 on NK cells using annexin V and 7-aminoactinomycin D staining as a marker for apoptosis and cell death, respectively (Fig. 1*D*). We observed no alterations in programmed cell death following 48 h of culture with GSK-J4; however, the HDAC inhibitors significantly increased apoptosis. This effect was even present following the culture of NK cells in the presence of very low concentrations of inhibitor, and therefore we chose not to further investigate the role of HDAC inhibitors (Fig. S2*c*). However, the previously identified capacity of GSK-J4 to suppress TNF-α production in stimulated human macrophages (16, 27) prompted us to focus on the effects of histone demethylase inhibition in NK cell cytokine production.

### Knockdown of the H3K27 demethylases JMJD3/UTX suppresses IFN-γ production in NK cells

**Figure 2.**
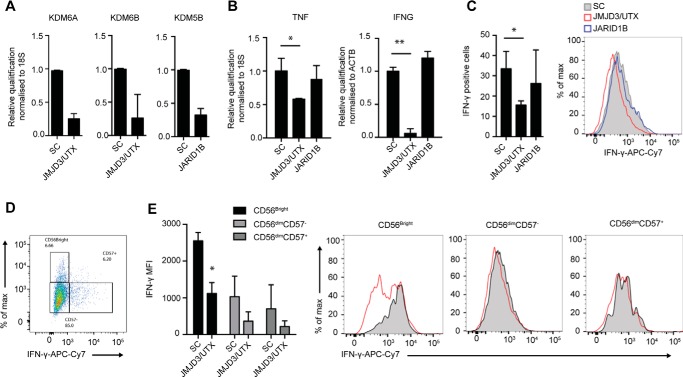
**Combined knockdown of KDM6A and KDM6B histone demethylases reduces IFN-γ and TNF-α production in human NK cell populations.** NK cells were cultured in the presence of IL-15 and JMJD3/UTX, JARID1B LNA target, and scrambled control (*SC*) oligonucleotides for 8 days. (*n* = 5). *A*, knockdown efficiency of targets was confirmed by measuring the levels of KDM5A (UTX), KDM5B (JARID1B), and KDM6B (JMJD3) by qPCR (*n* = 5). *B*, combined knockdown of JMJD3/UTX reduces the expression of TNF-α and IFN-**γ** mRNA levels, as confirmed by qPCR (*n* = 5). *C*, the expression of IFN-γ protein was measured by flow cytometry. NK cells were gated based on the expression of CD56 and CD57, followed by determination of intracellular IFN-γ (*n* = 3). *D*, a representative figure showing the gating strategy for CD56^bright^CD57^−^, CD56^dim^CD57^−^, and CD56^dim^CD57^+^ NK cell populations. *E*, the median fluorescence intensity of IFN-γ among CD56^bright^CD57^−^, CD56^dim^CD57^−^, and CD56^dim^CD57^+^ NK cell populations following knockdown of JMJD3/UTX as measured by flow cytometry. The *right panel* shows a representative flow cytometry histogram of IFN-γ expression in each NK cell population. The data are from three independent experiments. The data represent the means ± S.D. in *A–D*. *, *p* < 0.05; **, *p* < 0.01 (Kruskal–Wallis with Dunn's multiple comparison test). *MFI*, median fluorescence intensity.

GSK-J4 has been identified as a cell-permeable and potent inhibitor of KDM6 demethylases (16, 27) and has ∼5–20-fold lower activity against KDM5 demethylase enzymes (16, 42). To assess the specificity of the GSK-J4 compound on NK cell cytokine production, we used locked nucleic acid (LNA) knockdown of the H3K27 demethylases KDM6A/B (UTX and JMJD3, respectively) and H3K4me3 demethylase KDM5B (JARID1B), the targets for GSK-J4 (16, 42). LNA nucleotides are generated with a methylene bridge connecting the 2′ oxygen and 4′ carbon, resulting in increased stability and affinity and leading to efficient knockdown without the use of transfection or transduction techniques (43). To mimic the pan-KDM6 activity of GSK-J4, we used a dual JMJD3 and UTX LNA, which resulted in significant knockdown for both JMJD3 and UTX mRNA when compared with a scrambled control LNA, as confirmed by quantitative RT-PCR (Fig. 2*A*). Although KDM5B LNA treatment resulted in efficient reduction of its target (Fig. 2*A*), no effect on IFN-γ expression was observed (Fig. 2*B*). In contrast, knocking down JMJD3/UTX resulted in significantly reduced expression of both IFN-γ mRNA and protein expression following IL-15 stimulation (Fig. 2, *B* and *C*); furthermore, we also observed reduced expression of TNF-α mRNA expression following knockdown of JMJD3/UTX (Fig. 2*B*). Similar to using compound inhibition, the reduction in IFN-γ expression was also observed in three different NK cell subsets following LNA knockdown (Fig. 2, *D* and *E*). Collectively, these inhibition and knockdown results highlight a critical role for the Jumonji histone demethylases JMJD3 and UTX in regulating NK cell pro-inflammatory function.

### The demethylase inhibitor GSK-J4 reduces NK cell inflammatory functions

**Figure 3.**
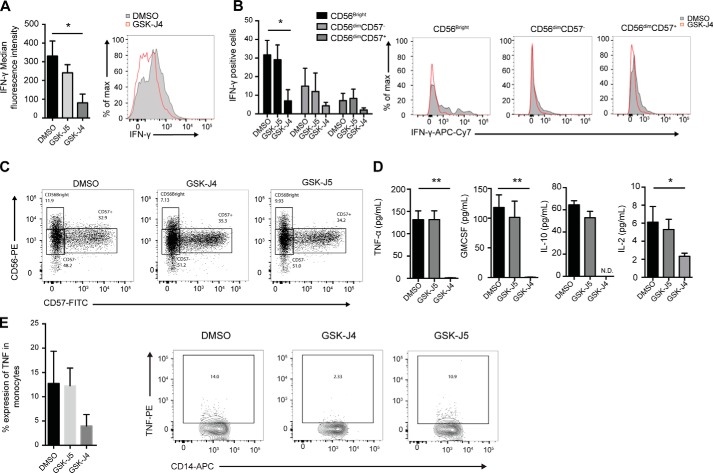
**GSK-J4 inhibits pro-inflammatory cytokine production in human peripheral blood NK cell subsets.** *A*, NK cells were isolated from peripheral blood and stimulated with IL-15 for 24 h. Protein transport inhibitor (brefeldin A/monensin) was added for the final 4 h of culture, before staining for CD56, CD57, and IFN-γ, prior to running through a flow cytometer. The data represent the means ± S.D. of three donors. The *right panel* shows a representative histogram of IFN-γ expression in one of three individual donors. *B*, the amount of IFN-**γ** was determined in CD56^bright^CD57^−^, CD56^dim^CD57^−^, and CD56^dim^CD57^+^ NK cell populations following 48 h of DMSO and GSK-J4 treatment as measured by flow cytometry. The data represent the means ± S.D. of three donors. The *right panels* show representative flow cytometry histograms of IFN-γ expression in each NK cell population. The data shown in the histograms represent one of three independent experiments. *C*, NK cells were isolated from peripheral blood and cultured in the presence of DMSO, GSK-J4, or GSK-J5 for 48 h and then stained with anti-CD56 and anti-CD57 antibodies. The frequencies of CD56^bright^CD57^−^, CD56^dim^CD57^−^, and CD56^dim^CD57^+^ were determined by flow cytometry. The data shown are representative of 14 donors. *D*, NK cells were isolated from peripheral blood, stimulated with IL-15, and then cultured for 48 h in the presence of DMSO or small molecule epigenetic inhibitors. The levels of secreted TNF-α, GM-CSF, IL-10, and IL-2 were measured. The data represent means ± S.D. of three donors. *E*, NK cells were isolated from peripheral blood, stimulated with IL-15, and then cultured in the presence of DMSO, GSK-J4, or GSK-J5 for 24 h. The cells were then washed twice in media and cultured in the presence of autologous CD14+ monocytes for a further 24 h. The cells were stained with CD14-APC and TNF-PE and then analyzed by flow cytometry. *, *p* < 0.05; **, *p* < 0.01 (Kruskal–Wallis with Dunn's multiple comparison test).

Given the complex regulation of pro-inflammatory cytokine secretion in NK cells, we next investigated whether reduced IFN-γ secretion was a consequence of an inhibition of cytokine mRNA expression or of impaired intracellular transport. Using flow cytometry, we found that GSK-J4, but not its inactive isomer control compound, GSK-J5, reduced NK cell–derived IFN-γ protein expression (Fig. 3*A*). We next investigated the relationship between IFN-γ production and GSK-J4 treatment in different peripheral blood-derived NK cell populations (Fig. 3*B*). Consistent with previous studies, CD56^Bright^ cells produce the highest levels of IFN-γ when compared with the other NK cell populations. Importantly, GSK-J4 treatment resulted in a reduced IFN-γ production in all NK cell subsets (CD56^Bright^CD57^−^, CD56^dim^CD57^+^, and CD56^dim^CD57^−^), whereas the control compound GSK-J5 had little effect. Moreover, GSK-J4 did not alter the NK cell population frequency, as assessed by flow cytometry (Fig. 3*C*).

In addition to IFN-γ, we also found that GSK-J4 led to a reduction in pro-inflammatory TNF-α, GM-CSF, IL-2, and anti-inflammatory IL-10 (Fig. 3*D*). In addition to producing intrinsic TNF-α, NK cells can also induce TNF-α production in CD14+ monocytes in a manner that is cell contact–dependent (44, 45). Therefore, we next investigated the ability of NK cells to induce macrophage-derived TNF-α in co-culture experiments. We found that NK cells pretreated for 24 h with GSK-J4 reduced the ability to induce TNF-α production in CD14+ co-cultures (Fig. 3*E*). Overall, these data demonstrate that GSK-J4 is able to regulate a diverse set of NK cell inflammatory functions.

### NK cell cytokine production from chronic inflamed tissue is reduced following GSK-J4 treatment

**Figure 4.**
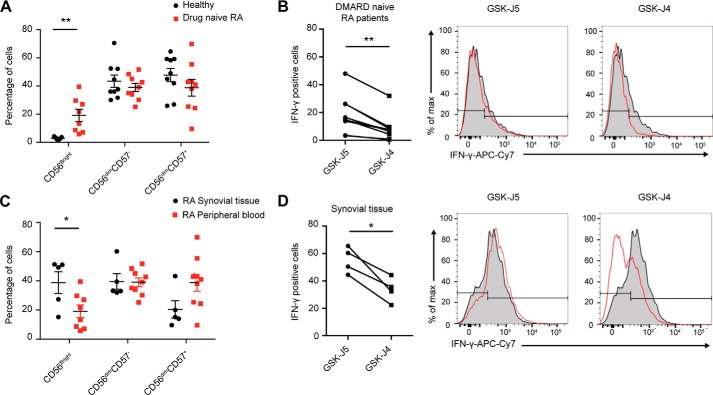
**GSK-J4 treatment reduces IFN-γ expression in NK cells isolated from the tissue of rheumatoid arthritis patients.** *A*, the frequency of CD56^bright^, CD56^dim^CD57^−^, and CD56^dim^CD57^+^ NK cell populations in peripheral blood from drug naïve RA patients and healthy individuals (*n* = 9). *B*, the frequency of IFN-γ^+^ NK cells isolated from RA patient peripheral blood. The *right panels* show representative histograms of IFN-γ expression. *C*, the frequency of CD56^bright^, CD56^dim^CD57^−^, and CD56^dim^CD57^+^ NK cell populations in synovial tissue samples and peripheral blood from RA patients. *D*, the frequency of IFN-γ^+^ cells among total NK cell populations isolated from RA synovial tissue. The *right panels* show representative histograms of IFN-γ expression. *A* and *C*, each *dot* represents a single sample, and a *horizontal bar* indicates the average for each group. The data shown are from nine healthy individuals, nine drug naïve RA patients, and five RA synovial tissue samples. *, *p* < 0.05; **, *p* < 0.01 (Mann–Whitney *U* test). *B* and *D*, *, *p* < 0.05; **, *p* < 0.01 (Wilcoxon matched-pairs test).

Having established a specific role for JMJD3/UTX H3K27 demethylases in reducing inflammatory cytokine levels, we next investigated these effects in a chronic inflammatory disease setting. In RA, NK cells are readily detectable in the synovial tissue of patients at an early disease stage and constitute ∼20% of all lymphocytes in the synovial fluid of established RA disease patients (46, 47). Characteristically, the synovial joint is enriched with CD56^Bright^ NK cells that secrete significantly higher levels of IFN-γ following IL-15 stimulation when compared with peripheral blood mononuclear cells from the same patient (46). We therefore used this chronic inflammatory disease as a model of inflammation to investigate the ability of GSK-J4 to reduce IFN-γ production. Interestingly, we observed a significant increase in the frequency of CD56^Bright^ cells in treatment-naïve RA patient peripheral blood when compared with healthy individuals (Fig. 4*A*). Mirroring the results from healthy NK cells, we observed a significant reduction in the frequency of IFN-γ when RA NK cells were treated with GSK-J4 (Fig. 4*B*). In agreement with literature (46), we also observed a significant increase in CD56^Bright^ NK cell frequency in RA synovial tissue explants when compared with treatment-naïve RA patient peripheral blood (Fig. 4*C*). When synovial NK cells were cultured in the presence of GSK-J4, a reduction in IFN-γ expression was also observed (Fig. 4*D*). Taken together, this suggests that in addition to reducing acute inflammation, GSK-J4 may also be beneficial in reducing inflammatory cytokine hallmarks in a situation of chronic inflammation.

### NK cell–mediated osteoclast formation is regulated by JMJD3/UTX

**Figure 5.**
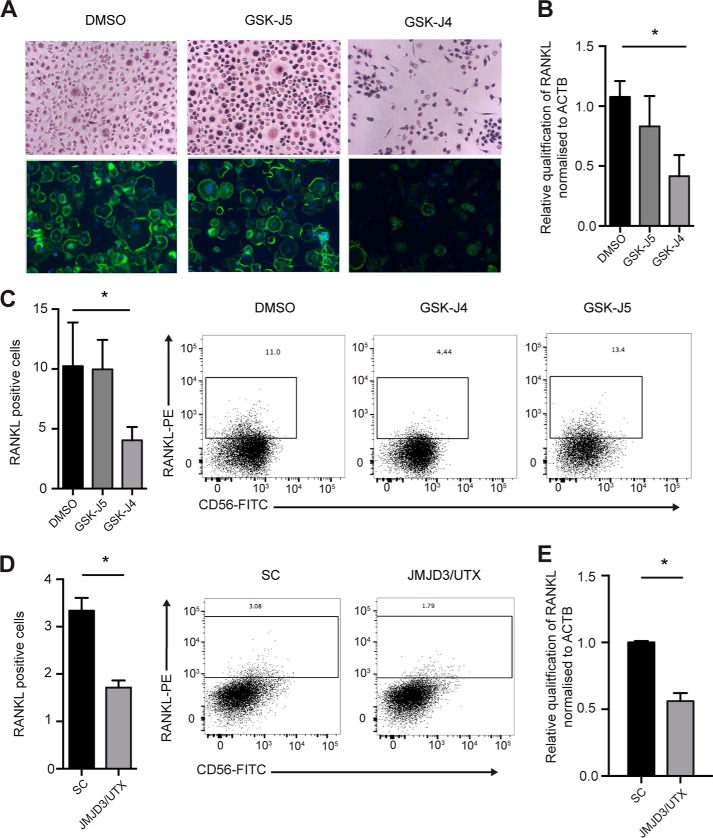
**NK cells cultured with GSK-J4 reduce RANKL expression resulting in inhibition of NK-mediated osteoclastogenesis.** *A*, CD56^bright^ NK cells were isolated from peripheral blood of RA patients and cultured in the presence of DMSO, GSK-J4, or GSK-J5 for 24 h, then washed in media, and cultured at a ratio of 1:5 with monocytes for 14 days. Osteoclast staining for TRAP (*purple*) (*top panels*) and immunofluorescence (*bottom panels*) was performed using DAPI (*blue*) and phalloidin (*green*) (20× magnification) (*n* = 5). *B*, NK cells were isolated from RA peripheral blood, stimulated with IL-15, and then cultured in the presence of DMSO, GSK-J4, and GSK-J5 for 72 h. The expression of RANKL was determined using RT-PCR. *C*, protein expression was determined using flow cytometry. The data represent means ± S.D. of five donors. The *right panels* show representative histograms of three independent donors. *, *p* < 0.05 (Kruskal–Wallis with Dunn's multiple comparison test). *D*, NK cells were isolated from peripheral blood, stimulated with IL-15, and then cultured in the presence of locked nucleic acid targeted toward both JMJD3 and UTX. Surface expression of RANKL on NK cells was measured by flow cytometry. *E*, expression of mRNA was determined by RT-PCR. The data represent the means ± S.D. of five donors. *, *p* < 0.05 (Mann–Whitney test).

The data obtained thus far indicated a role for H3K27 demethylases in reducing inflammatory cytokine output in NK cells. However, NK cells from RA patients are also involved in mediating osteoclastogenesis, leading to bone erosion (4). Therefore, we were interested to identify whether Jumonji demethylase inhibition could impact this phenotype. To address this question, we treated CD56^bright^ NK cells isolated from the peripheral blood of RA patients and cultured them in the presence of DMSO, GSK-J4, or GSK-J5 for 24 h. NK cells were washed and then co-cultured in the presence of allogeneic monocytes. Following 14 days of culture, the presence of multinucleated osteoclasts was determined. We found that NK cell pretreatment with GSK-J4 resulted in significantly reduced osteoclast numbers (Fig. 5*A*). A key factor for monocyte fusion and osteoclast formation is RANKL (*receptor activator for nuclear factor kappa b ligand*); accordingly we tested whether NK cell RANKL expression was altered upon GSK-J4 treatment. Indeed, RANKL expression was reduced both in RA patient and healthy donor-derived NK cells following GSK-J4 treatment, as determined by RT-PCR and flow cytometry (Fig. 5, *B* and *C*). This effect is specific to knockdown of KDM6A and KDM6B, as evidenced by down-regulation of RANKL expression using the LNA knockdown approach (Fig. 5, *D* and *E*). These data suggest that GSK-J4 can inhibit osteoclastogenesis by reducing the expression of RANKL on the surface of NK cells.

### The transcriptional profile of NK cells is altered following inhibition of JMJD3/UTX H3K27 demethylases

**Figure 6.**
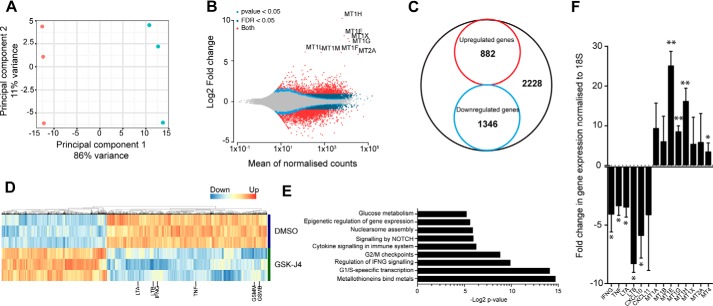
**Transcriptional changes in NK cells isolated from peripheral blood in response to GSK-J4 treatment.** *A*, principal component analysis of treated samples. *Red*, control (DMSO) treatment; *blue*, GSK-J4 treatment following RNA-seq analysis. *B*, MA plot summarizing differential expression in NK cells following GSK-J4 treatment. *C*, Venn diagram showing proportions of significantly changed (log2-fold) genes upon GSK-J4 treatment. *D*, heat map of gene expression in three individual NK cell donors upon GSK-J4 treatment. *E*, biological classification of significantly changed genes. *F*, validation of selected genes by RT-PCR analysis. The data represent the means ± S.D. of nine donors. ANOVA was determined by Tukey multiple comparison test. *, *p* < 0.05; **, *p* < 0.01.

The evident phenotypic consequences of inhibiting the histone H3K27 demethylase activity on inflammatory NK cell function prompted us to investigate global transcriptional changes upon GSK-J4 treatment. Expression analysis was performed by RNA sequencing (RNA-seq) in IL-15–stimulated NK cells following a 15-h GSK-J4 treatment. These data reveal a transcriptional signature that is comprised of 2200 genes with significant log2-fold changes, of which ∼60% are down-regulated (Fig. 6, *A–D*) using a false discovery rate of 0.05. A pathway enrichment analysis of the differentially regulated genes (supporting information) shows that GSK-J4 impacts on several processes, comprising cell cycle, cytokine production, growth factor regulation, signaling, epigenetic, and chromatin pathways, as well as metabolic pathways (Fig. 6*E*). This is in line with our previous observations showing an anti-inflammatory and anti-proliferative effect following GSK-J4 treatment. Whereas pathways such as Notch signaling and glycolysis have been previously shown to be important for NK cell function (48, 49), we observed a strong induction of metallothionein genes; however, the reason for this remains enigmatic at this point. Next, we validated the RNA-seq data by performing qPCR on NK cells treated for 24 h with GSK-J4. This confirmed a large metallothionein up-regulation and a reduction of inflammatory cytokines IFN-γ, TNF-α, LTA, and LTB (Fig. 6*F*), demonstrating a robust NK cell transcriptomic change following H3K27 demethylase inhibition.

### NK cell cytolytic function is unaffected following JMJD3/UTX-driven H3K27 demethylation

**Figure 7.**
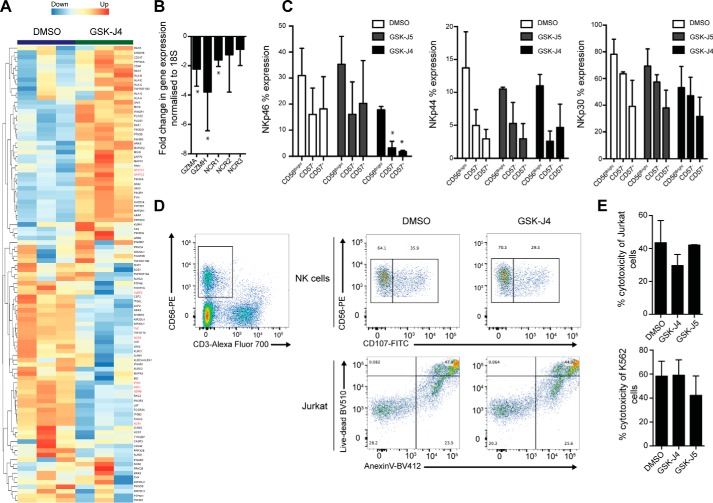
**GSK-J4 inhibition leads to selective changes in genes associated with NK cell–mediated cell lysis but does not impair tumor cell killing *ex vivo*.** *A*, heat map of NK cell genes associated with lytic functions after treatment with GSK-J4 for 24 h. Genes discussed in the text are highlighted. *B*, qPCR confirmation of selected NK cell effector genes. The data represent the means ± S.D. of nine donors. *, *p* < 0.05 (ANOVA with Tukey multiple comparison test). *C*, flow cytometry analysis of activating NK cell receptors (NCR1, NCR2, and NCR3) in different NK cell populations after GSK-J4 treatment. The data represent the means ± S.D. of four donors. *, *p* < 0.05 (ANOVA with Tukey multiple comparison test). *D*, gating strategy and flow cytometry of NK cell killing assays using Jurkat cell lines. The data are representative of three independent donors. *E*, cell lysis of Jurkat (*upper panel*) or K562 (*lower panel*) measured by LDH release upon GSK-J4 or GSK-J5 treatment. The data represent the means ± S.D. of three donors.

Having identified that GSK-J4 inhibition leads to a strongly altered inflammatory profile in NK cells, we next asked whether this could impact on the cytolytic function of NK cells. Selecting genes that are associated with NK cell cytolytic function and tumor cell killing revealed an informative pattern of gene expression upon GSK-J4 treatment. In addition to the above mentioned inflammatory cytokines, the transcriptomic data also revealed down-regulation of genes involved in NK cell–mediated killing, such as granzyme (GZMB), perforin, NCRs, and UL16-binding proteins (ULBPs) (Fig. 7*A*). On the other hand, up-regulated genes following GSK-J4 treatment represent signal transduction components such as IFN receptors, various kinases, and phosphatases, as well as transcription factors (NFATc1 and NFATc2) (Fig. 7*A*).

Next, we confirmed this transcriptional pattern by selecting targets from the list of genes and performing qPCR and flow cytometry analysis. This analysis revealed that NKp30 (NCR3), NKp44 (NCR2), and NKp46 (NCR1), as well as granzymes (GZMB and GZMH), had expression patterns mirroring that of the RNA-seq data (Fig. 7*B*). However, protein expression levels revealed that only NKp46 was significantly reduced following either 48 h of GSK-J4 treatment or JMJD3/UTX knockdown in various NK cell populations, as shown by flow cytometry (Fig. 7*C* and Fig. S3).

The significantly altered transcriptional response for genes involved in NK cell killing led us to next examine whether functional NK cell killing was impaired. This was investigated by using two different types of established NK cell killing assays: the LDH release and the CD107 assay (50), with two different cancer cell lines used as targets for killing (Jurkat and K562). This revealed no significant differences in the ability of NK cells to kill target cells following GSK-J4 treatment (Fig. 7, *D* and *E*, and Fig. S3*d*). Despite the significant transcriptional change, the impact of a 48-h GSK-J4 treatment on protein expression appears modest and shows that GSK-J4 inhibition has no significant impact on the ability of human NK cells to mediate killing of cancer cells in these *ex vivo* systems.

### A global increase in histone H3K27 methylation results in reduced NK cell inflammatory function

**Figure 8.**
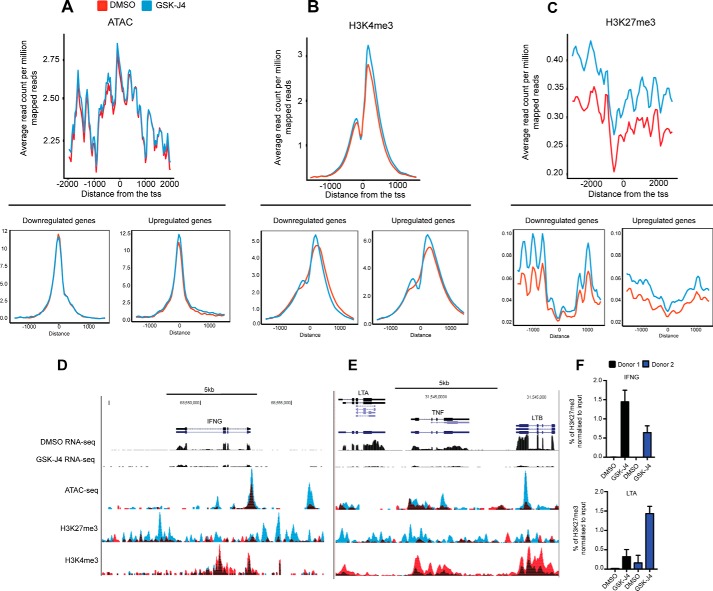
**Genome-wide changes in H3K4me3 and H3K27me3 enrichment following NK cells treated with GSK-J4.** *A*, average coverage of ATAC-seq reads around the TSS of all reference genes following 24 h of DMSO and GSK-J4 treatment. Coverage plots showing the reads from ATAC-seq over the down-regulated (*bottom left panel*) and up-regulated genes (*bottom right panel*) are also shown. *B* and *C*, average H3K4me3 and H3K27me3 coverage across the TSS of all reference genes following 24 h of DMSO and GSK-J4 treatment. Coverage plots showing the reads from H3K27me3 and H3K4me3 over the down-regulated (*bottom left panel*) and up-regulated genes (*bottom right panel*) are also shown. *D* and *E*, representative RNA-seq and ChIP-seq coverage profiles of H3K4me3, H3K27me3 across the *IFNG* locus, and the *TNF* locus. For each gene, the coverage profile was normalized by dividing the average coverage in that gene. *Red*, DMSO; *blue*, GSK-J4-treated. *F*, the ChIP-PCR results confirm the ChIP-seq results at the IFNG and LTA promoters. The data shown are for two donors with triplicate measurements each.

Next we wanted to investigate the molecular mechanism involved in inducing the transcriptional change observed following GSK-J4 treatment. Using the assay for transposable–accessible chromatin sequencing (ATAC-seq) method to interrogate chromatin accessibility, we observed no significant global alterations following treatment of NK cells with GSK-J4 (Fig. 8*A*). However, when looking at profiles across the promoters of down-regulated and up-regulated genes (as determined in Fig. 6), we observe an enrichment in up-regulated genes in cells treated with GSK-J4. Intriguingly, visual inspection of selected gene loci, such as that of IFN-γ, TNF-α, and LTA/LTB, also show a local increase in chromatin accessibility, despite a clear down-regulation in mRNA expression (Fig. 8*D*).

Next, to investigate the targets of the Jumonji histone demethylases, we performed chromatin immunoprecipitation followed by sequencing (ChIP-seq) with antibodies against H3K4me3 and H3K27me3 histone marks. When the level of enrichment of H3K27me3 across all transcriptional start sites (TSSs) of the genome is considered, GSK-J4 treatment leads to a global increase in H3K27me3 levels, with a small increase in global H3K4me3 levels (Fig. 8, *B* and *C*). Interestingly, this pattern between the down-regulated and up-regulated genes is consistent for both marks, indicating complex global changes in histone marks upon inhibitor treatment. However, upon visual inspection of representative GSK-J4 inflammatory target genes, such as LTA, TNF-α, LTB, and IFN-γ, we observe significantly increased levels of the repressive H3K27me3 mark (Fig. 8, *D* and *E*). This was accompanied by a concomitant decrease in permissive H3K4me3 levels, reflecting the observed reduction in RNA and protein levels seen previously (Fig. 6). Using ChIP-PCR, confirmation of the levels of enrichment for H3K27me3 was performed at the promoter sites of IFN-γ and LTB (Fig. 8*F*). Taken together, these genome-wide epigenomic experiments reveal a significant redistribution and global increase of repressive H3K27 marks that could explain the observed down-regulation of critical NK-cell effector genes.

## Discussion

The epigenetic mechanisms underlying the generation and maintenance of functional NK cells remain only partially understood. It is known that a number of transcription factors such as STAT4, STAT5, and T-bet are responsible for the expression of NK cell effector genes such as perforin, IFN-γ, and TNF-α (51). Despite this, the contribution of chromatin factors that control the NK cell transcriptional circuitry is largely undetermined. Genetic analysis of a rare type of non-Hodgkin lymphoma, the extranodal NK/T-cell lymphoma nasal type (ENKL), highlights the general importance of chromatin modification and remodeling factors including BCL6 co-repressor, the methyltransferase EZH2 and SUZ12, components of polycomb repressor complex 2 (PRC2), for NK cell pathophysiology (52). Among the epigenetic factors regulating non-malignant NK cell biology, DNA methylation plays an important role in the regulation of both IFN-γ and perforin expression following IL-15 stimulation (32). Similarly, it has been shown that NK cell function is also regulated by histone acetylation, because HDAC inhibitors suppress IFN-γ expression following cytokine stimulation (53), a finding that is fully confirmed by our screening data with a subset of experimental and clinical HDAC inhibitors. Although our results suggest a more cytotoxic role for this inhibitor class, it is possible that HDAC inhibitors also suppress NK cell cytotoxic function through down-regulation of NK cell activation receptors NKp30, NKp46, and NKG2D (53, 54). Our finding that BET bromodomain inhibitors potently down-regulate IFN-γ production further supports the role of chromatin acetylation and recognition of acetyl-lysine by reader domains in NK cell biology.

In terms of histone lysine methylation, only a few recent studies have highlighted the importance of this modification in NK cell function. An inhibitory role of the H3K27 methyltransferase EZH2 in the development of CD122^+^ NK cells from hematopoietic precursors was postulated, with a concomitant increase of NKG2D receptor expression upon EZH2 knockdown (35), confirming the role of H3K27 methylation in lymphocyte lineage development. Genetic ablation of the histone H3K4me3 demethylase KDM5A (JARID1A) leads to modulation of NK cell function by down-regulating IFN-γ production as a consequence of impaired STAT4 signaling and increased levels of SOCS1 (suppressor of cytokine 1) (55), an effect that was also observed by reduction of IFN-γ production in the Nishi NK-like cell line (Fig. S1) using the pan-KDM5 inhibitor KDM5-C70 (56).

Our study now adds important novel aspects to the understanding of how histone H3K27 modification regulates NK cell functions. First, the coinciding phenotypes of chemical inhibition of H3K27 demethylases UTX and JMJD3 (KDM6A and KDM6B, respectively) validated with their simultaneous LNA knockdown profiles clearly establish a role of histone H3K27 methylation in NK cell cytokine regulation. The phenotypic consequences of cytokine repression are best explained by the global increases of H3K27 methylation upon GSK-J4 treatment, as demonstrated by the quantitative ChIP-seq data and furthermore reflected in the significant increases of this mark around the transcriptional start sites of IFNG, TNF, and other inflammatory genes. Although not shown in our work, this increase in repressive H3K27 marks presumably leads to an increased binding of PRC1 (57), thus effectively down-regulating gene transcription. However, the gene silencing mediated by PRC proteins is undoubtedly more complex and not fully understood (49), because the observed global increase in H3K27me levels in NK cells does not lead to a global decrease in gene transcription, suggestive of an intricate selectivity mechanism for PRC–mediated repression in NK cells.

Furthermore, the observed increases in gene transcription of glycolytic genes upon GSK-J4 treatment (Fig. 6 and supporting information) highlight a recently uncovered complex connection between metabolic regulation and cytokine production in NK cells. In contrast to resting lymphocytes, cytokine-activated lymphocytes, including NK cells, undergo a metabolic switch to a significantly elevated rate of aerobic glycolysis (58). This is accompanied by increased expression of glycolytic enzymes and nutrient transporters (59). This increased flux through the glycolytic pathway, controlled by mTORC1 signaling, presumably provides the necessary metabolic precursors for proliferation and effector functions (60). Interestingly, enhanced glycolysis supports discrete NK cell effector functions, shown by glycolysis inhibition experiments demonstrating down-regulation of IFN-γ and perforin secretion, which is dissociated from a TNF-α response (59, 61). Our KDM6 inhibition data provide another layer of complexity in regulating NK cell biology, showing increased RNA levels of glycolytic enzymes, whereas in general, effector molecules are reduced without alteration of direct cytotoxicity effector functions.

Like other lymphoid and myeloid immune cell populations, NK cells apparently display a high degree of plasticity, reflecting tissue origin and disease exposure (62). In all our investigated NK cell subsets, isolated from either peripheral blood or RA tissue, GSK-J4 inhibition leads to an IFN-γ suppression, irrespective of the tissue of origin. Moreover, other cytokines including TNF-α, GM-CSF, and anti-inflammatory IL-10, although only investigated in blood-derived NK cells, appear to be down-regulated in a similar manner as IFN-γ, suggesting a similar repressive mechanism. Of particular interest is the expression of RANKL found in the NK cell population of RA patients, leading to an increased osteoclast formation with concomitant bone erosion, a hallmark of inflammatory disease in RA (4). The observed inhibition of RANKL expression by GSK-J4 in this pro-inflammatory NK cell subset, leading to a decrease in osteoclastogenesis, thus lends credence to the concept of a general anti-inflammatory phenotype of KDM6 inhibition in human immune cells (16, 27).

Whereas our data highlight the importance of reversible histone H3K27 methylation/demethylation in NK cell function, the question of a therapeutic utility of KDM6 inhibition in the contexts of autoimmunity, infection, and immuno-oncology remains an open one. The initial discovery of the anti-inflammatory effects of KDM6 inhibition in human stimulated macrophages (16, 27) and our data on healthy and RA–derived NK cells provides evidence that it might be possible to find a therapeutic window for beneficial inflammatory modulation.

## Experimental procedures

### NK cell preparation and cell culture experiments

All of the cell lines used in this study were regularly tested for mycoplasma infection by RT-PCR. NK cells were isolated from venous blood, obtained from healthy volunteers, or from single-donor platelet pheresis residues from the Oxford National Health Service Blood Transfusion Service. Peripheral blood was obtained from RA patients attending the rheumatology clinic at Northwick Park Hospital, London (approved study, Research Ethics Committee (REC) Number 07/H0706/81). The human biological samples were sourced ethically, and their research use was in accord with the terms of the informed consents. The patients were diagnosed according to the American College of Rheumatology (ACR) Eular 2010 criteria. Mononuclear cells were isolated by Ficoll density gradient centrifugation and CD56^+^ NK cells were isolated from peripheral blood mononuclear cells by negative isolation using Dynabeads (Invitrogen). NK cell populations were cultured in IMDM (Gibco) supplemented with 5% heat-inactivated human serum, in the presence of 50 ng/ml IL-15 at 37 °C in 5% CO_2_. For isolation of monocytes, the monocyte isolation kit (Miltenyi) was used. Monocytes were cultured for 2 days in RPMI 1640 (Gibco) supplemented with 5% heat inactivated human serum in the presence of inhibitors or controls. For osteoclast differentiation, co-culture experiments with monocytes and NK cells were carried out. The cells were washed three times in IMDM before being resuspended in IMDM supplemented with 10% human serum and cultured at a 1:5 ratio of monocytes to NK cells. For patient cells, NK cells were isolated directly from peripheral blood using Dynabeads. For direct comparison, blood was obtained from healthy volunteers and isolated in parallel. Following NK cell and monocyte culture for 14 days, the cells were washed three times with 200 μl of PBS, and adherent cells were fixed with 4% paraformaldehyde in PBS for 5 min. After washing in water, multinucleated osteoclasts were stained for tartrate-resistant acid phosphatase (TRAP) as described (4). TRAP^+^ multinucleated giant cells were quantified by processing the digital images using ImageJ. Immunofluorescence staining was performed following the TRAP assay, and the cells were washed twice in PBS, permeabilized in methanol at −20 °C for 2 s, washed twice in PBS, and washed once in PBS-BSA. Saponin (0.2% in PBS) was then added to the cells for 10 min. The permeabilized cells were then incubated for 30 min with fluorescein-conjugated phalloidin (20 μm), washed twice in PBS-BSA, and washed once in PBS. The cells were visualized using a Zeiss confocal microscope. In each experiment, pictures from different samples were taken consecutively using identical microscope settings.

### NK cell–mediated cancer cell killing assays

1 × 10^6^ NK cells were stimulated with IL-15 (50 ng/ml, Peprotech) and cultured in the presence of GSK-J4 or controls for 24 h. The cells were washed twice in fresh medium before they were added to the killing assay. In 96-well round-bottomed plates, the target cells were incubated in 200 μl of medium with 1 × 10^5^ pretreated NK cells at an effector–target ratio of 5:1 for 4 h. Assay medium was phenol red-free X-VIVO 15 supplemented with 10% human serum. The cancer cells used were JURKAT (clone E6–1, ATCC), a T lymphocyte cell line, and K562 (clone CCL-234, ATCC), a myelogenous leukemic cell line. 50 μl/well of supernatant was then transferred to a 96-well plate, and lysis was analyzed by measuring lactate dehydrogenase release using the Cytotox 96 assay kit (Promega), according to the manufacturer's instructions. Controls to measure spontaneous LDH release from target and effector cells were included. The percentage of lysis was calculated according to the manufacturer's instructions using the formula: ((Experimental − Effector_Spontaneous_ − Target_Spontaneous_)/(Target_Maximum_ − Target_Spontaneous_)) × 100. NK cell degranulation assays were performed as previously described (50). Briefly, NK cells were cultured with target cells at a 1:1 ratio in the presence of the anti-CD107a antibody (H4A3, Biolegend) and incubated for 1 h at 37 °C. Protein transport inhibitor (mixture of brefeldin A/monensin, eBiosciences) was then added, and the cells were incubated for 4 h at 37 °C. After staining with anti-CD56 antibody, annexin V, and the addition of 7-AAD, surface expression of CD107a was assessed by flow cytometry on a Becton Dickinson LSR Fortessa^TM^ instrument. Data analysis was performed using the FlowJo program.

### Cytokine secretion assay

NK or Nishi cells (39) cells were seeded at 200,000 cells/well in 200 μl of culture medium in 96-well plates. Compounds were dissolved in DMSO at concentrations described in Table S1 and diluted to achieve the desired working solutions. Compound effects were compared with cells cultured in 0.1% DMSO alone, whereas wells filled with media served as a background control. Following 24 h of stimulation with IL-15 (50 ng/ml, Peprotech), IFN-γ secretion was determined in the cell culture supernatant by ELISA (eBioscience) according to the manufacturer's instructions. CellTitre 96® cell proliferation assay (MTS) was used to determine proliferation, according to the manufacturer's instructions. Cell viability was determined by hemocytometer cell counting. For electrochemiluminescence studies, the human pro-inflammatory-9 ultra-sensitive kit from Mesoscale Discovery measuring IL-6, IL-8, IL-10, TNF-α, IL12p70, IL-1β, GM-CSF, IL-2, and IFN-γ was used. Mesoscale Discovery plates were analyzed on the MS2400 imager (Mesoscale Discovery). The assay was performed according to the manufacturer's instructions, and all standards and samples were measured in triplicate.

### Flow cytometry analysis

Cell surface marker expression was analyzed on a Becton Dickinson LSR Fortessa^TM^ flow cytometer after staining with fluorochrome-conjugated antibodies. Intracellular staining was performed using the fix/perm buffer set (Biolegend), according to the manufacturer's instructions. Antibodies used were conjugated anti-NKp30 (P30–15, Biolegend), anti-NKp44 (P44–8.1, BD), anti-NKp46 (29A1.4, Biolegend), anti-IFN-γ (485.B3, Biolegend), anti-TNF-α (MAb11, eBioscience), anti-RANKL (MIH24, Biolegend), anti-Ki-67 (16A8, Biolegend), anti-CD107a (H4A3, BD), anti-CD57 (HCD57, Biolegend), anti-CD56 (N901, Beckman Coulter), anti-CD14 (HCD14, Biolegend), anti-CD16 (3G8, Biolegend), and anti-CD3 (UCHT1, Biolegend). Annexin V/7-AAD staining was performed according to the manufacturer's protocol (Biolegend). The data were analyzed using FlowJo v10.2 software.

### Quantitative RT-PCR

First-strand cDNA synthesis was achieved by reverse transcription of RNA using iScript^TM^ reverse transcription supermix (Bio-Rad). cDNA served as template for amplification of genes of interest and housekeeping genes by RT-PCR, using SYBR Green MasterMix (Applied Biosystems) amplified and read using the ViiA^TM^ 7 real-time PCR machine. The primers used in this study are listed in Table S3. The *C*t values were normalized using RPLPO, ACTB, and 16S as internal controls.

### Locked nucleic acid–mediated knockdown experiments

Knockdown experiments were conducted using locked nucleic acids (LNA) antisense oligonucleotides designed and synthesized as described previously (43) by Santaris A/S. Following synthesis oligonucleotides were purified by HPLC, desalted using a Milliprep membrane, and verified by LC-MS. NK cells were cultured at a concentration of 1 × 10^6^ cells/ml and stimulated with IL-15, and LNAs were added to the culture medium at a concentration of 1 μm and then cultured for a further 8 days.

### RNA isolation and RNA-seq library preparation

RNA was isolated from NK cells using the Quick-RNA MiniPrep kit (Zymo) according to the manufacturer's protocol. The quality of the RNA samples was verified by electrophoresis on Tapestation (Agilent). The RIN scores for all samples were in the range of 7.5–9.5.

RNA-seq libraries were prepared using the NEBNext® Ultra^TM^ RNA library prep kit for Illumina® using TruSeq indexes, according to the manufacturer's protocol. The resulting libraries were sequenced on a NextSeq 500 platform (Illumina) using a paired-end run 2 × 80 bp, to an average depth of 112 × 10^6^ paired-end reads/sample (range 47 × 10^6^ to 168 × 10^6^).

### ChIP-seq and ChIP-PCR

Per chromatin immunoprecipitation experiment, 30 × 10^6^ total NK cells were used. For H3K27me3 ChIP, three independent donors were pooled prior to sonication. All other ChIP-seq assays were performed using single donors. For quantitative ChIP experiments, 4 × 10^6^ SF1 cells were spiked into the pool. The cells were then cross-linked by formaldehyde treatment, and chromatin was fragmented to 200–300 bp by sonication using a Biorupter® Pico (Diagenode). Each lysate was immunoprecipitated with 10 μg of the following antibodies: H3K4me3 (Merck Millipore) and H3K27me3 (Merck Millipore). The ChIP was then performed for each antibody as described previously by Orlando *et al.* (63). Purified DNA was used for library preparation using a NEBNext Ultra DNA sample preparation kit (NEB) according to the manufacturer's recommendations. The samples were multiplexed, quantified using a Kapa library quantification kit (KAPA Biosystems), and sequenced on a NextSeq 500 (Illumina) platform (paired-end, 2 × 41 bp for H3K4me3 and 2 × 80 bp for H3K27me3). Sequencing depth was in excess of 20 million reads/sample, suggesting sufficient coverage.

For ChIP-PCR, the following primer pairs were used to amplify the IFNG and LTA promoter sites by PCR. The primers used for IFNG were 5′-CTC TGG CTG GTA TTT AT-3′ (forward) and 5′-CAT CCC TGC CTA TCT GT-3′ (reverse), and those for LTA were 5′-GCC TTT GAC TGA AAC AGC A-3′ (forward) and 5′-GCC TTT GAC TGA AAC AGC A-3′ (reverse). PCR amplification was performed with 2 μl of DNA recovered from ChIP in a total volume of 10 μl or reaction mixture containing 100 nm of each primer and 5 μl of SYBR fast master mix (Applied Biosystems). The reactions were performed with 40 cycles of denaturing at 95 °C for 3 s and anneal/extension at 60 °C for 30 s using the ViiA^TM^ 7 PCR system (Applied Biosystems).

### Assay for transposable–accessible chromatin using sequencing

ATAC-seq was performed using 100,000 cells for the transposition reaction, which was performed as described by Buenrostro *et al.* (64) using in-house produced transposase. Subsequently, the samples were purified using the GeneJET PCR purification kit (Thermo). PCR amplification was performed using the following protocol: 3 min at 72 °C, 30 s at 98 °C and 11 cycles of 10 s at 98 °C, 30 s at 63 °C, and 3 min at 72 °C. The samples were then purified using the GeneJET PCR purification kit and eluted with 20 μl of TE buffer. Samples were then validated on a Tapestation (Agilent) to determine library size and quantification prior to paired-end (2 × 41 bp) sequencing on a NextSeq 500 (Illumina) platform.

### Processing of next-generation sequencing data

A computational pipeline was written calling scripts from the CGAT toolkit to analyze the next generation sequencing data (65) and (https://github.com/CGATOxford/CGATPipelines).^4^ For RNA-seq experiments, sequencing reads were mapped to the reference human genome sequence (GRCh37 (hg19) assembly) using hisat v0.1.6 (66). To count the reads mapped to individual genes, the program featureCounts v1.4.6 (67) was used. Only uniquely mapped reads were used in the counting step. The counts table that was generated was then used for differential expression analysis. Differential gene expression analysis was performed with DESeq2 v1.12.3 within the R statistical framework v3.3.0 (68). To define differentially expressed genes, a threshold of >2-fold change, and a false discovery rate of <0.05 were used.

For analysis of ChIP-seq and ATAC-seq data, Bowtie software v0.12.5 was used to align the reads to the human hg19 reference genome (69). Reads were only considered that were uniquely aligned to the genome with up to two mismatches. For quantitative ChIP, the number of reads mapping to SF9 cells was determined for each sample. Bedtools version 2.2.24 was used to generate bedgraph files from the mapped BAM files using the scaling factor derived from the SF9 read count. Averaged tracks for each condition were produced representing the mean of the scaled values for biological replicates. Homer tag directories were then produced using the raw read function, and coverage plots around the TSS were plotted in R. MACS software (v1.4.2) or SICER (v1.1) was used to identify enrichment of intervals of H3K4me3 and H3K27me3 following ChIP-seq and regions of open chromatin following ATAC-seq. Sequencing of the whole cell extract was performed to determine the background model when analyzing ChIP-seq. For ATAC-seq, peak callers were run without a background. Enrichment of reads around the TSS and enhancer sites was calculated and plotted across the genome using the ngs.plot.r package (70). For visualization as a University of California Santa Cruz Genome Browser track, the bam files generated from Bowtie were converted to BigWig files using bam2wiggle (65).

### Statistical analysis

All values are presented as means ± S.D. Mann–Whitney *U* test and Kruskal–Wallis with Dunn's tests were used for multiple comparisons. Wilcoxon matched-pairs test was used when analyzing paired samples. *p* values lower than 0.05 were considered significant. All calculations were performed in R and GraphPad prism. The sample size was chosen based on previous work in macrophages (16).

In the figures *asterisks* indicate *p* values as follows: *, *p* < 0.05; **, *p* < 0.01. For RNA-seq, the Padj values were used to determine significance and are Tables S1–S3. In all situations, the control and the treatment conditions were prepared and analyzed simultaneously from the same donor.

### Data availability

RNA-seq, ATACs-eq, and ChIP-seq data sets are deposited with the GEO database under accession number GSE89669.

## Author contributions

A. C. and U. O. conceptualization; A. C., E. S. H., and U. O. formal analysis; A. C., G. W., A. S., M. P., and H. P. investigation; A. C., M. L., S. O., H. O., R. K. P., N. A., K. S., and M. F. methodology; A. C. and U. O. writing-original draft; A. C. and U. O. writing-review and editing; M. L., S. O., H. O., and R. K. P. resources; N. A., M. F., and U. O. funding acquisition; U. O. supervision.

## Supplementary Material

Supporting Information

## Footnotes

4 Please note that the JBC is not responsible for the long-term archiving and maintenance of this site or any other third party hosted site.

## Footnotes

3 The abbreviations used are:

NK: natural killer
IFN: interferon
TNF: tumor necrosis factor
IL: interleukin
RA: rheumatoid arthritis
HDAC: histone deacetylase
LNA: locked nucleic acid
RNA-seq: RNA sequencing
qPCR: quantitative PCR
ATAC-seq: assay for transposable-accessible chromatin using sequencing
ChIP-seq: chromatin immunoprecipitation followed by sequencing
TSS: transcriptional start site
PRC: polycomb repressor complex
GM-CSF: granulocyte–macrophage colony-stimulating factor
IMDM: Iscove's modified Dulbecco's medium
TRAP: tartrate-resistant acid phosphatase
ChIP-PCR: chromatin immunoprecipitation followed by PCR
ANOVA: analysis of variance
BET: bromodomain and extra-terminal family protein
LTA: lymphotoxin A
LTB: lymphotoxin B
NCR: natural cytotoxicity receptor
MTS: 3-(4,5-dimethylthiazol-2-yl)-5-(3-carboxymethoxyphenyl)-2-(4-sulfophenyl)-2*H*-tetrazolium.
