# Supplementary material for: Inhibition of histone H3K27 demethylases selectively modulates inflammatory phenotypes of natural killer cells
Source: J Biol Chem. 2018 Jan 4;293(7):2422–37. doi: 10.1074/jbc.RA117.000698 (PMC5818173; doi:10.1074/jbc.RA117.000698)
Supplement: Supporting Information [file supp_293_7_2422__index.html]

­­­Inhibition of histone H3K27 demethylase selectively modulates inflammatory phenotypes of natural killer cells — Epigenetic modulation of NK cell phenotypes — Inhibition of histone H3K27 demethylases selectively modulates inflammatory phenotypes of natural killer cells — Epigenetic modulation of NK cell phenotypes — Supporting Information 

# Inhibition of histone H3K27 demethylases selectively modulates inflammatory phenotypes of natural killer cells

## Supporting Information

- Supplementary Information - SI Data Cribbs et al
- SI Material - SI Material Cribbs et al- regulated genes
