# Supplementary material for: Inhibition of histone H3K27 demethylases selectively modulates inflammatory phenotypes of natural killer cells
Source: J Biol Chem. 2018 Jan 4;293(7):2422–37. doi: 10.1074/jbc.RA117.000698 (PMC5818173; doi:10.1074/jbc.RA117.000698)
Supplement: Supporting Information [file supp_RA117.000698_133519_1_supp_42996_p1bs8z.pdf]

SUPPLEMENTARY MATERIAL

Selective modulation of inflammatory Natural Killer (NK) cell phenotypes following histone H3K27 demethylase inhibition

Adam Cribbs, Edward S Hookway, Graham Wells, Morten Lindow, Susanna Obad, Henrik Oerum, Rab Prinjha, Nick Athanasou, Aneka Sowman, Martin Philpott, Henry Penn, Kalle Soderstrom, Marc Feldmann, Udo Oppermann

TABLE OF CONTENTS

SUPPLEMENTARY FIGURES

Supplementary Figure 1: Results of epigenetic compound screen for IFN-γ production in the Nishi NK cell line.

Supplementary Figure 2: IC50 determination for IFNγ release in human NKcells treated with BET bromodomain or HDAC inhibitors.

SUPPLEMENTARY FIGURE 3: FACS analysis for NK receptors under GSK-J4 treatment

SUPPLEMENTARY TABLES

Supplementary Table 1: List and details of epigenetic tool compounds used in the initial IFN-γ screen.

Supplementary Table 2: Clinical details of RA patients

Supplementary Table 3: Sequences of RT-PCR primer pairs for genes used in this study

SUPPLEMENTARY FIGURES

SUPPLEMENTARY FIGURE 1: Results of epigenetic compound screen for IFN-γ production in the Nishi NK cell line.

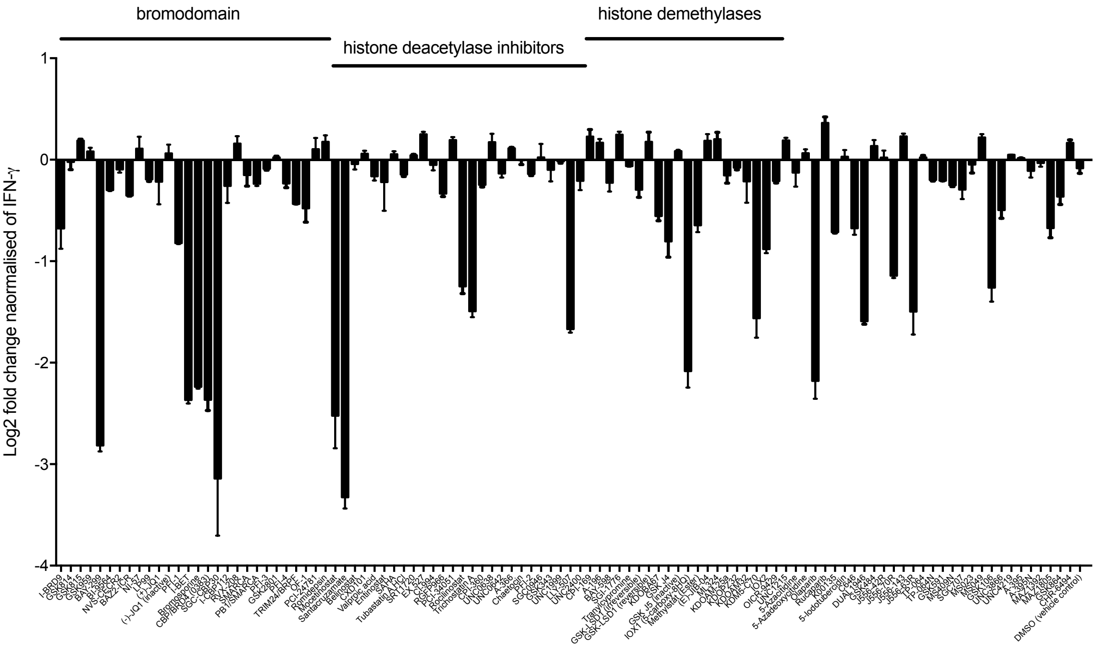

Nishi cells were stimulated with IL-15 then cultured for 24 hours in the presence of DMSO or small molecule epigenetic inhibitors and supernatant was harvested. Levels of IFN-γ were measured by ELISA and log2 fold changes were determined against DMSO.

SUPPLEMENTARY FIGURE 2: IC50 determination for IFNγ release in human NKcells treated with BET bromodomain or HDAC inhibitors.

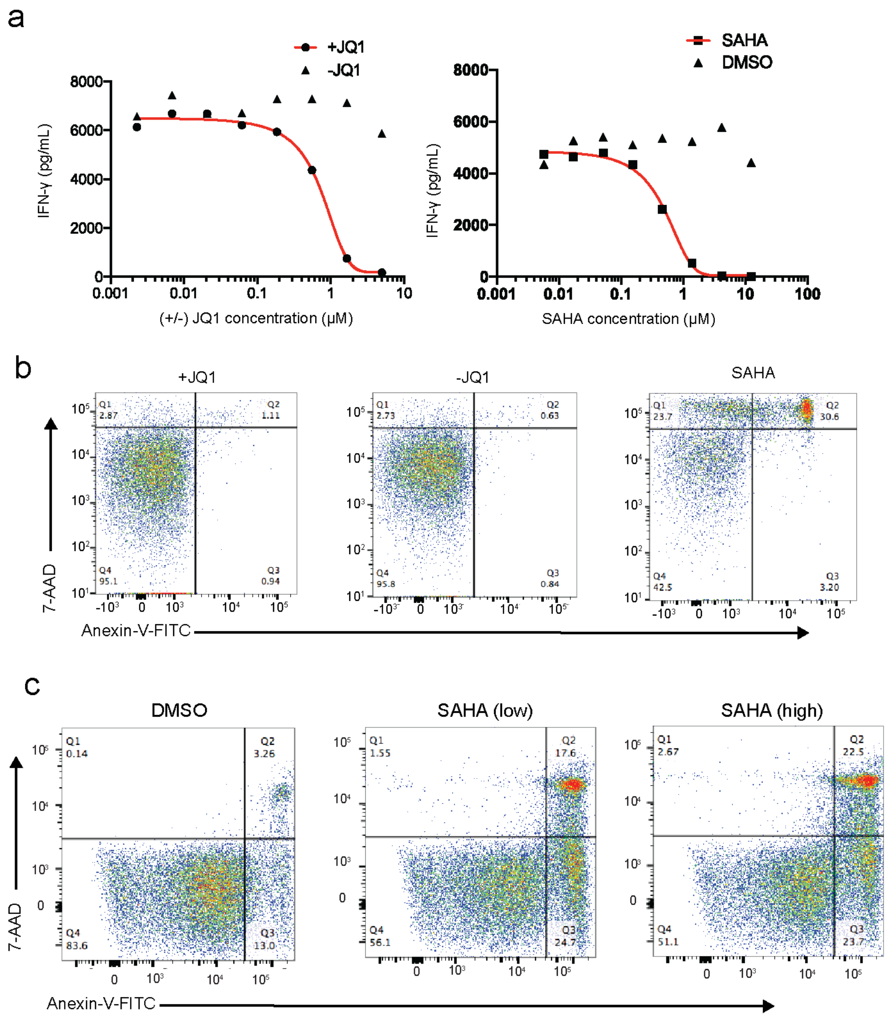

NK cells were isolated from peripheral blood, stimulated with IL-15 then cultured for 48 hours in the presence of DMSO or small molecule epigenetic inhibitors and supernatant was harvested. (A) The IC50 of +JQ1 and SAHA was determined for each inhibitor with IL-12 and IL-15 stimulated NK cells following 48 hours of culture. (B) NK cells were isolated from peripheral blood, stimulated with IL-15 and cultured in the presence of DMSO, +JQ1 and –JQ1 for 48 hours. Cells were stained with Anexin-V-FITC and 7-AAD then analysed using flow cytometry. In each panel the lower left quadrant shows cells, which are negative for both 7-AAD and Anexin-V, upper left quadrant shows only 7-AAD positive cells, which are necrotic. The lower right quadrant shows cells that are positive for Anexin-V (early apoptotic) and the upper right shows Anexin-V and 7-AAD positive cells (late apoptotic). (C) NK cells were cultured in 0.25 μM (Low) and 2.5 μM (High) concentrations of SAHA for 48 hours then stained with Anexin-V-FITC and 7-AAD then analysed using flow cytometry.

SUPPLEMENTARY FIGURE 3: FACS analysis for NK receptors under GSK-J4 treatment

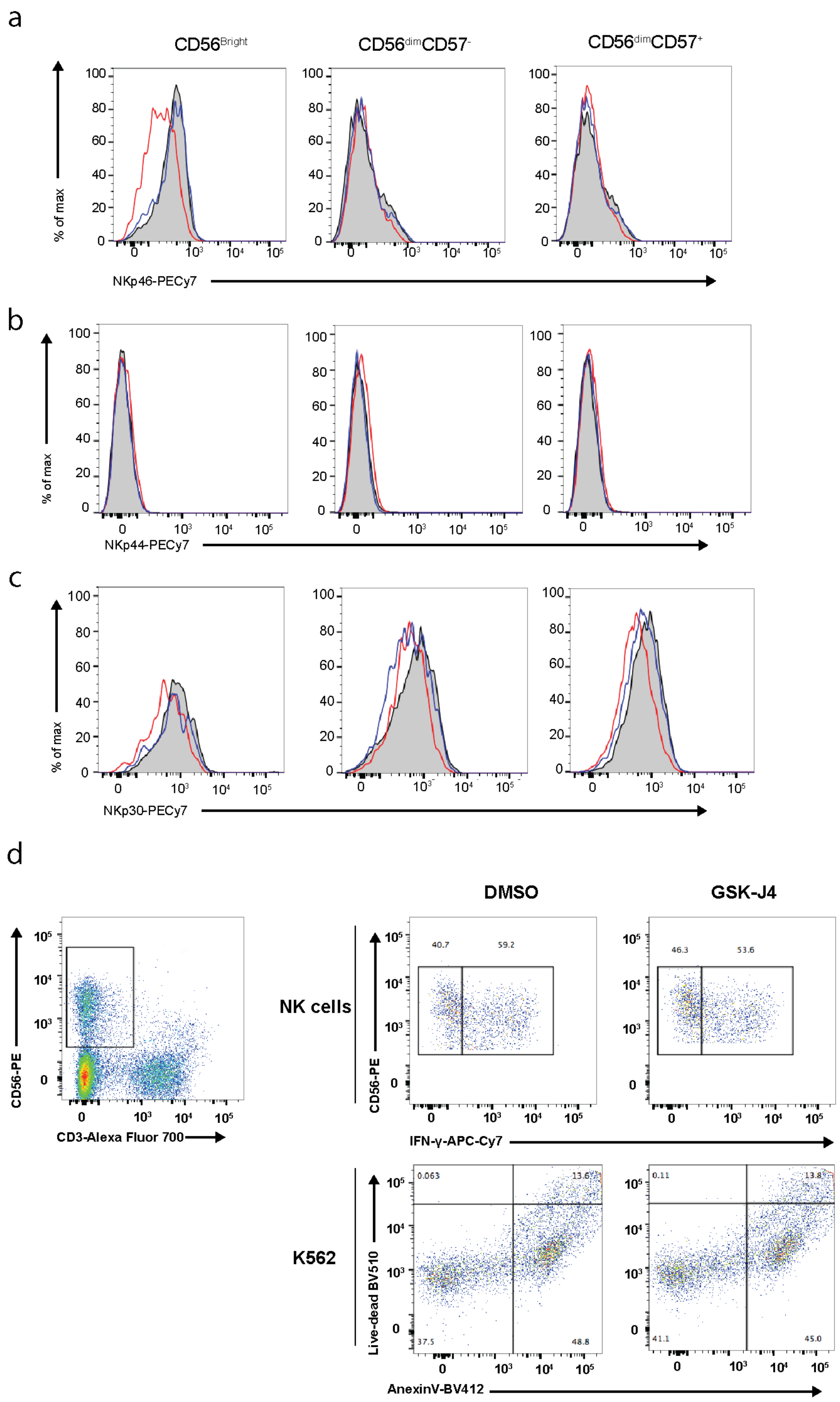

(a, b, c) Flow cytometry analysis of activating NK cell receptors (NCR1, NCR2, NCR3) in different NK cell populations after GSK-J4 treatment. Data shows representative histogram of four independent experiments. (d) Gating strategy and flow cytometry of NK cell killing assays using K562 cell lines. Data is representative of three independent donors.

SUPPLEMENTARY TABLES

Supplementary Table 1: List and details of epigenetic tool compounds used in the initial IFN-γ screen. Major target classes are highlighted by colour coding.

CLASS	TARGET	COMPOUND NAME	SCREENING CONCENTRATION [μM]	SUPPLIER	PubMed ID	COMMENTS
Arginine methyltransferase	PRMT3	SGC707	1	Sigma	25728001	
Arginine methyltransferase	PRMT4	TP-064	1	SGC		
Arginine methyltransferase	PRMT4	TP-064N	1	SGC		inactive control for TP-064
Arginine methyltransferase	PRMT4, PRMT6	MS049	1	Sigma	27584694	
Arginine methyltransferase	PRMT4, PRMT6	MS409N	1	Sigma	27584694	inactive control for MS409
Arginine methyltransferase	PRMT5	GSK591	1	Sigma	26985292	
Arginine methyltransferase	PRMT5	J556-143	1	Changchun Discovery Sciences		
Arginine methyltransferase	PRMT5	J556-42R	1	Changchun Discovery Sciences		
Arginine methyltransferase	PRMT5	J556-63R	1	Changchun Discovery Sciences		
Arginine methyltransferase	PRMT5	J556-70R	1	Changchun Discovery Sciences		
Arginine methyltransferase	Type I PRMTs	MS023	1	Sigma	26598975	
Bromodomains	ATAD2	GSK814	10	GSK		
Bromodomains	ATAD2	GSK815	10	GSK		inactive control for GSK814
Bromodomains	BAZ2A, BAZ2B	BAZ2-ICR	1	Sigma	25719566	
Bromodomains	BAZ2A, BAZ2B	GSK2801	1	SGC	25799074	
Bromodomains	BRD1, TAF1	BAY-299	1	SGC		
Bromodomains	BRD2, BRD3, BRD4, BRDT	(-)-JQ1	1	Sigma	20871596	inactive control for (+)-JQ1
Bromodomains	BRD2, BRD3, BRD4, BRDT	(+)-JQ1	1	Sigma	20871596	
Bromodomains	BRD2, BRD3, BRD4, BRDT	I-BET	1	Sigma	21068722	
Bromodomains	BRD2, BRD3, BRD4, BRDT	PFI-1	5	Sigma	23576556	
Bromodomains	BRD2, BRD3, BRD4, BRDT	RVX-208	5	Cayman	24248379	
Bromodomains	BRD9	I-BRD9	10	Sigma	25856009	
Bromodomains	BRD9, BRD7	BI-9564	1	Sigma	26914985	
Bromodomains	BRD9, BRD7	LP99	1	Sigma	25864491	
Bromodomains	BRPF (pan)	NI-57	1	Sigma		
Bromodomains	BRPF (pan)	OF-1	5	Sigma		
Bromodomains	BRPF1	GSK959	1	GSK		
Bromodomains	BRPF1B	PFI-4	1	Sigma		
Bromodomains	CECR2	NVS-CECR2-1	1	Cayman		
Bromodomains	CECR2	NVS-CECR2-C	1	Cayman		inactive control for NVS-CECR2-1

Bromodomains	CREBBP, BRD4	BDOIA000383	5	SGC	24946055	
Bromodomains	CREBBP, EP300	I-CBP112	1	Sigma	26552700	
Bromodomains	CREBBP, EP300	SGC-CBP30	1	Sigma	26261308	
Bromodomains	pan-Bromodomain	Bromosporine	1	Sigma	27757418	
Bromodomains	SMARCA2, SMARCA4, PBRM1	PFI-3	1	Sigma	26702435	
Bromodomains	SMARCA2, SMARCA4, PBRM1	BDF00008718	2.5	SGC		
Bromodomains	SMARCA2, SMARCA4, PBRM1	BDF00021522		SGC		
Bromodomains	TRIM24, BRPF	BDOCA000405a	10	SGC	25974391	
Other – Chromodomain	CBX4, CBX7	UNC3866	30	UNC	26807715	
Other – Chromodomain	CBX4, CBX8	UNC4219	30	UNC	26807715	inactive control for UNC3866
Other – Dehydrogenase	IDH1 R132C/R132H/R132G	GSK864	5	Sigma	26436839	
DNA methyltransferase	DNMT1, DNMT3	5-Azacytidine	10	Sigma	18425818	
DNA methyltransferase	DNMT1, DNMT3	5-Azadeoxycytidine	5	Sigma	18425818	
Histone acetyltransferase	p300/CREBBP	C646	1	Sigma	21518915	
Histone deacetylase	Class I	CI-994	1	Selleckchem	15839304	
Histone deacetylase	Class I	Romidepsin	1	Selleckchem	12208741	
Histone deacetylase	Class I & II	Valproic acid	1000	Sigma	11742974	
Histone deacetylase	Class I, II & IV	Belinostat	5	Selleckchem	12939461	
Histone deacetylase	Class I, II & IV	SAHA	2.5	Cambridge Bioscience	11016644	
Histone deacetylase	Class I, II & IV	Trichostatin A	0.5	Sigma	11309348	
Histone deacetylase	Class I, IIB	PCI-24781	10	Selleckchem	16731764	
Histone deacetylase	HDAC1, HDAC3	Entinostat	0.5	Selleckchem	10200307	
Histone deacetylase	HDAC1,HDAC2, HDAC3, HDAC11	Mocetinostat	10	Selleckchem	18413790	
Histone deacetylase	HDAC2	Santacruzamate	50	Selleckchem	24164245	
Histone deacetylase	HDAC3	RGFP966	10	Selleckchem	23894374	
Histone deacetylase	HDAC6	Rocilinostat	10	Selleckchem	22262760	
Histone deacetylase	HDAC6	Tubastatin A HCl	10	Selleckchem	20614936	
Histone deacetylase	HDAC8	PCI-34051	5	Selleckchem	18256683	
Histone deacetylase	SIRT1	EX 527	1	Selleckchem	16354677	
Histone deacetylase	SIRT1	SRT1720	1	Selleckchem	18046409	activator
Histone methyltransferase	DOT1L	SGC0946	7.5	Sigma	23250418	
Histone methyltransferase	EZH2	GSK343	3	Sigma	24900432	
Histone methyltransferase	EZH2	UNC1999	1	Sigma	23614352	
Histone methyltransferase	EZH2	UNC2400	1	Sigma	23614352	inactive control for UNC1999
Histone methyltransferase	EZH2, EZH1	CPI-169	10	Selleckchem	25457180	
Histone methyltransferase	EZH2, EZH1	CPI-360	10	Selleckchem	24183969	
Histone methyltransferase	G9a, GLP	A-366	2	Sigma	24900801	
Histone methyltransferase	G9a, GLP	UNC0638	1	Sigma	21743462	
Histone methyltransferase	G9a, GLP	UNC0642	1	Sigma	24102134	
Histone methyltransferase	SETD7	PFI-2	2	Sigma	25136132	
Histone	SMYD2	BAY-598	1	Sigma	27075367	

methyltransferase						
Histone methyltransferase	SMYD2	LLY-507	1	Sigma	25825497	
Histone methyltransferase	SUV39H1	Chaetocin	0.05	Sigma	16408017	
Histone methyltransferase	SUV420H1, SUV420H2	A-196	1	Sigma		
Other - Kinase inhibitor	Haspin	5-Iodotubercidin	1	Selleckchem	23071153	
Other - Kinase inhibitor	Haspin	CHR-6494	1	Sigma	21804608	
Other - Kinase inhibitor	Haspin	SGI-1776	10	Selleckchem	19734450	
Other - Kinase inhibitor	PIM	K00135	1	SGC	17638903	
Lysine demethylases	JmjC (pan)	(E)-JIB-04	0.05		23792809	
Lysine demethylases	JmjC (pan)	Methylstat (Ester)	2.5		21585201	
Lysine demethylases	KDM4	ML324	5	(NIH/NCATS)	24260783	
Lysine demethylases	KDM5	KDM5-C70	10	Xcess Biosciences	27214403	
Lysine demethylases	KDM5	KDOAM000025	1	SGC		
Lysine demethylases	KDM5	KDOAM000032	1	SGC		inactive control for KDOAM000025
Lysine demethylases	KDM5	KDOPZ000032	1	SGC		
Lysine demethylases	KDM5B, KDM6A, KDM6B	GSK-J4	10	Tocris	22842901	
Lysine demethylases	KDM5B, KDM6A, KDM6B	GSK-J5	10	Tocris	22842901	inactive control for GSK-J4
Lysine demethylases	KDM5B, KDM6A, KDM6B	KDOBA000067	10	SGC		
Lysine demethylases	LSD1	GSK690	5		24912735	
Lysine demethylases	LSD1	GSK-LSD1	0.5	Sigma		
Lysine demethylases	LSD1	Tranylcypromine	20	Sigma	16793513	
Lysine demethylases	pan-2-OG	IOX1	40	Sigma	21124847	
Other- Methyl Lysine Binder	EED	A-395	1	SGC		
Other- Methyl Lysine Binder	EED	A-395N	1	SGC		inactive control for A-395
Other- Methyl Lysine Binder	L3MBTL3	UNC1215	5	Sigma	23292653	
Other- Methyl Lysine Binder	WDR5	OICR-9429	1	SGC	26167872	
Other- Peptidyl arginine deiminase	PAD4	GSK106	1	Sigma	25622091	inactive control for GSK484
Other- Peptidyl arginine deiminase	PAD4	GSK484	1	Sigma	25622091	
Other- Poly ADP ribose polymerase	PARP1	Rucaparib	10	Stratec Scientific	17363489	
Other- Poly ADP ribose polymerase	PARP1, PARP2	Olaparib	1	Selleckchem	18800822	
Other- Prolyl-Hydroxylases	PHD2	IOX2	10	SGC	23683440	

Supplementary Table 2: Clinical details of RA patients providing samples to this study.

(A) Clinical details of rheumatoid arthritis (RA) patients providing blood samples used in this study. All samples were taken on the day of diagnosis and patients were drug naïve. Abbreviations: anti-CCP: anti-cyclic citrullinated peptides; RF: rheumatoid factor; DAS28: Disease activity score 28; n.a. = not available

Sex	Age	Anti-CCP	RF	DAS28	Smoker
Female	60	+	+	5.16	No
Female	48	+	-	5.94	Prev
Male	43	+	+	4.27	No
Female	72	+	+	4.45	No
Female	28	+	+	5.00	No
Female	44	+	+	4.60	No
Female	50	+	+	5.13	Prev
Female	60	n.a.	+	7.50	No

(B) Details for RA patients providing RA synovial tissue. Patients were previously diagnosed and treated. Abbreviations: MTX: methotrexate; n.a. = not available

Sex	Age	Treatment	RF
Female	65	MTX	+
Female	72	MTX	n.a.
Female	56	MTX	n.a.
Female	75	MTX	n.a.
Female	79	MTX	n.a.
Female	72	MTX, sulfasalazine	n.a.

Supplementary Table 3: Sequences of RT-PCR primer pairs for genes used in this study

Gene	Forward primer	Reverse primer
IFNG	TCGGTAACTGACTTGAATGTCCA	TCGCTTCCCTGTTTTAGCTGC
TNF	GAGGCCAAGCCCTGGTATG	CGGGCCGATTGATCTCAGC
LTA	CATCTACTTCGTCTACTCCCAGG	CCCCGTGGTACATCGAGTG
LTB	GGAGACGACGAAGGAACAGG	GTAGAGGTAATAGAGGCCGTCC
CXCL10	GTGGCATTCAAGGAGTACCTC	TGATGGCCTTCGATTCTGGATT
CXCL11	GACGCTGTCTTTGCATAGGC	GGATTTAGGCATCGTTGTCCTTT
GZMA	ATTCTTGGGGCTCACTCAATAAC	GGGTCATAGCATGGATAGGGAAA
GZMH	CTGGCTGGGGTTATGTCTCAA	GGCTACGTCCTTACACACGAG
NCR1	TGGACCCGAAGTGATCTCG	TCCTTGAGCAGTAAGAACATGC
NCR2	GGCTCTCAGGCACAATCCAAG	GCTGAAGCCTCCTTACACCA
NCR3	CCCCTGAGATTCGTACCCTG	CTCCACTCTGCACACGTAGAT
MT1A	GTGCGCCTTATAGCCTCTCA	AGGAGCAGCAGCTCTTCTTG
MT1B	TCCTGCAAGAAGTGCTGCTG	GCAAACCGGTCAGGGTAGTT
MT1E	CGCCACTGGTGGCTCC	AAGAGCTGTTCCCACATCAGG
MT1G	TCCTGCAAGAAGAGCTGCTG	TTTGTACTTGGGAGCAGGGC
MT1H	CCCTCCACGTGTTCCACTG	AGGAGCAGCAGCTCTTCTTG
MT1X	ACCTCCTGCAAGAAGAGCTG	TAGCAAACGGGTCAGGGTTG
MT4	CACTGGAGCCTTTCGGACAC	TGCAGATTCCTCCAGACATGC
MT2A	TCCTCCAAGTCCCAGCGAAC	GAGCAGCAGCTTTTCTTGCAG
